# Anti-Cholinesterase Combination Drug Therapy as a Potential Treatment for Alzheimer’s Disease

**DOI:** 10.3390/brainsci11020184

**Published:** 2021-02-02

**Authors:** Hafsa Amat-ur-Rasool, Mehboob Ahmed, Shahida Hasnain, Wayne G. Carter

**Affiliations:** 1 Royal Derby Hospital Centre, School of Medicine, University of Nottingham, Derby DE22 3DT, UK; hafsa.phd.mmg@pu.edu.pk; 2 Department of Microbiology and Molecular Genetics, University of the Punjab, Lahore 54590, Pakistan; mehboob.mmg@pu.edu.pk (M.A.); Shahida.mmg@pu.edu.pk (S.H.)

**Keywords:** Alzheimer’s disease, cholinesterase inhibitors, drug synergism

## Abstract

Alzheimer’s disease (AD) is a burgeoning social and healthcare problem. Cholinesterase inhibitors (ChEIs) are employed for symptomatic treatment of AD, but often elicit adverse drug reactions (ADRs). Herein, the potency of the ChEIs, donepezil, tacrine, berberine, and galantamine to inhibit human or *Torpedo californica* acetylcholinesterase (*tc*AChE) proteins were evaluated. The efficacy of dual-drug combinations to inhibit human AChE directly and within differentiated neurons was also quantified. ChEI potency was in the order: donepezil > tacrine > berberine > galantamine for both AChEs. Dual-drug combinations of berberine and tacrine (BerTac), berberine and galantamine (BerGal), and tacrine and donepezil (TacDon) all produced synergistic outcomes for AChE inhibition. Donepezil and berberine (DonBer) and tacrine and galantamine (TacGal) elicited antagonistic responses. Donepezil and galantamine (DonGal) was synergistic for human AChE but antagonistic for *tc*AChE. After application of dual-drug combinations to neuronal cells, BerTac, BerGal, DonGal, and DonBer all showed synergistic inhibition of AChE, TacDon additive, and TacGal antagonistic effects. BerGal produced the most potent synergism and reduced total drug dose by 72%. Individual ChEIs or dual-drug combinations were relatively non-toxic to neuronal cells, and only reduced cell viability at concentrations two–three orders of magnitude greater than that required to inhibit AChE. In summary, dual-drug combinations of ChEIs potentially represent a novel means of AD patient treatment, with reduced and more cost-effective drug dosing, and lowered likelihood of ADRs.

## 1. Introduction

Approximately 50 million people globally suffer from dementia and the number is expected to triple by 2050 [1]. Alzheimer’s disease (AD) represents the majority of dementia cases [2,3]. AD patients suffer from broad symptomology that includes confusion, periods of memory loss and cognitive decline, and with disease progression, AD patients lose the ability to perform daily activities and tasks with an associated loss of independence [4]. The reduced cognition aligns with a loss of neuronal transmission and functionality, as well as a loss of neuronal mass [5,6]. AD patients experience brain cortical atrophy primarily within the frontotemporal lobes, notably within the hippocampal and entorhinal cortex [5,6], consistent with the loss of cognitive function and memory attributed to these brain regions [7].

A reduction of signaling from the neurotransmitter acetylcholine (ACh) led to the concept of a “cholinergic hypothesis” to explain some of the cognitive deficits experienced by AD patients [8,9]. This cholinergic signaling decline provides the basis for the current first-line drug treatment for mild-to-moderate AD: employment of cholinesterase inhibitors (ChEIs) such as donepezil, rivastigmine, and galantamine [10,11,12]. ChEIs transiently inhibit the activity of acetylcholinesterase (AChE), which is predominantly localized to the pre-synaptic membrane in the CNS and the synaptic basal lamina of the neuromuscular junction [13,14]. This (reversible) inhibition of AChE within the central nervous system (CNS) is therefore an attempt to prolong ACh signaling.

ChEIs are either of synthetic (e.g., donepezil and tacrine) or of plant origin (e.g., galantamine, huperzine A, and berberine). The current US Food and Drug Administration (FDA) approved ChEIs (donepezil, galantamine, and rivastigmine) have proved beneficial for patients with mild-to-moderate AD [15,16]; however, they are limited by induction of undesired side effects, and ultimately, only provide symptomatic rather than curative benefit. Nevertheless, the search continues for useful next-generation ChEIs with similar efficacy to the current drugs but with fewer adverse drug reactions (ADRs). Furthermore, additional beneficial properties such as provision of antioxidants, has garnered the use of certain plant extracts and phytochemicals as potential next-generation ChEIs [17,18,19,20,21,22].

To achieve efficacy, treatment with ChEIs often requires titration, typically with an increased drug dose after four weeks [15]. At the starting or increased drug dose there is still a risk of idiosyncratic ADRs. Furthermore, for chronically dosed patients, therapeutic effects may be limited (at a fixed dosing regimen) due to neurobiological adaptation and drug tolerance, promoting the need for further increased dosing. Therefore, the lowest drug dose possible that still achieves therapeutic efficacy will reduce the likelihood of induction of toxicity.

One approach to provide suitable drug efficacy to treat AD, but at a lower dosing regimen, is via combination drug therapy [23]. Combination drug therapy can provide a fixed-dose of two or more active pharmaceutical agents within a single dosing entity. In this study, the efficacy of individual ChEIs as well as two-drug combinations were assessed via their ability to directly inhibit AChE. The FDA-approved synthetic ChEIs, donepezil and tacrine, were investigated, as well as two plant alkaloids (phytochemicals), galantamine (also FDA approved) and berberine. Berberine was included as it is a phytochemical with documented AChE affinity and binding, antioxidant properties, and proposed as a potential anti-AD drug [24,25,26].

## 2. Materials and Methods

### 2.1. Chemicals and Reagents

Recombinant human acetylcholinesterase (hAChE), *Torpedo californica* acetylcholinesterase (*tc*AChE), acetylthiocholine iodide (ATCI), 5,5’-dithiobis-(2-nitrobenzoic acid) (DTNB), all-trans-retinoic acid, thiazolyl blue tetrazolium bromide (MTT), isopropanol, dimethyl sulfoxide (DMSO), and the drugs donepezil hydrochloride, MW = 415.95 g/mol; tacrine hydrochloride, MW = 234.72; berberine hydrochloride, MW = 371.82 g/mol; and galantamine hydrobromide, MW = 368.27 g/mol, were all purchased from Sigma-Aldrich (Poole, UK). Dulbecco’s Modified Eagle’s Medium (DMEM), fetal bovine serum (FBS), albumin, and poly-D-lysine (PDL) were purchased from Thermofisher Scientific (Rochester, UK). Unless specified differently, all other chemicals were from Sigma-Aldrich (Poole, UK).

### 2.2. Enzymatic Activity Assays

A microtiter plate version of the Ellman’s assay was used to determine the activity of human and *Torpedo californica* acetylcholinesterase enzymes [27]. Assays were undertaken in the presence of either individual drugs or two-drug combinations. Each assay data point within a 96-well plate contained 40 µL of 0.01 M DTNB, 46 µL of 0.1 M (pH 8.0) phosphate buffer, 2 µL of 0.075 M ATCI substrate, and 10 µL of drug or dual-drug combination. Drug concentrations were prepared according to Supplementary Table S1 [28]. Enzymatic cleavage of the ATCI substrate was initiated by addition of 2 µL of 0.5 U/mL AChE enzyme. The reaction was undertaken for 30 min at room temperature, protected from the light, before the color change was read at 412 nm using a Multiskan Spectrum plate reader (Thermo Electron Corporation, Vantaa, Finland). Under these assay conditions, a final color change was reached (results not included) such that an end-point assay measurement was taken [29].

Initially, assays were undertaken with a broad drug concentration range (0.1, 1, 10, 100 and 1000 µM) to determine an approximate concentration producing 50% inhibition of AChE (IC_50_) (results not included). These scoping experiments enabled us to quantify an approximate IC_50_ for subsequent dose-response experiments. Two-fold serial dilutions at the approximate drug IC_50_ concentrations were prepared i.e., 0.25×, 0.5×, 1×, 2×, and 4× the IC_50_ concentrations. These concentrations were also used for dual-drug combination experiments. Experiments undertaken in the absence of inhibitor were designated as 100% enzymatic activity. Assays were performed in duplicates from which a mean was calculated.

#### AChE Assay Using Neuroblastoma Cells

Neuroblastoma SH-SY5Y cells (*passage* #15) were grown in DMEM containing 10% FBS in 6-well plates that had been pre-coated with 50 µg/mL PDL. Cells were grown to ≈50% confluency at 37 °C within an atmosphere of 5% CO_2_ and 95% humidity. Growth media was replaced and cells differentiated in DMEM/F12 media (without choline or phenol red) containing 1% FBS and 10 µM all-trans-retinoic acid. Growth media was changed every other day and differentiation of neurons was monitored by light microscopy. After 6 days, neurons were fully differentiated, and then treated with drugs across the broad concentration range of 0.1, 1, 10, 100, and 1000 µM; either as individual drugs or in two-drug combinations. The cell-based Ellman’s assay described by Li et al. (2017) [29] was modified for 6-well plates. A final reaction volume of 400 µL was used, comprised of 190 µL of 0.01 M DTNB, 200 µL of 0.1 M (pH 8.0) phosphate buffer and 10 µL of 0.075 M ATCI substrate. AChE present on the cell membranes of the differentiated neuronal cells provided the enzymatic activity to drive the hydrolysis of ATCI. Plates were placed in the 37 °C cell culture incubator for 30 min. The reaction mixture was then removed from the plate wells and transferred into 1.5 mL Eppendorf tubes. These were centrifuged at 12,000× *g* for 5 min to settle cellular debris. The supernatant was removed and optical density measured at a wavelength of 412 nm using a Multiskan Spectrum plate reader (Thermo Electron Corporation, Vantaa, Finland).

### 2.3. Computational Analysis

Percentage inhibition of AChE from in vitro assays in response to application of individual or dual-drug ChEIs were calculated using CompuSyn software (http://www.combosyn.com/index.html). The CompuSyn report provided IC_50_ and combination index (CI) values for individual drug or two-drug combinations and was used to plot dose-response effect curves and CI plots, respectively [30].

### 2.4. Cytotoxicity Assays

A MTT assay was used to assess the cytotoxicity of either the individual drugs or dual-drug combinations using the SH-SY5Y neuroblastoma cell line. SH-SY5Y cells were seeded in 96-well plates (passage #15) at a cell density of 3 × 10^4^ cells/well in DMEM containing 10% FBS. Cells were grown until 80–85% confluent and then treated with drugs over a concentration range of 0.01, 1, 10, 100, and 1000 µg/mL in cell culture media. After an incubation of 48 h within the 37 °C incubator, cell culture media was removed and replaced with fresh growth media containing 0.5% (*w*/*v*) MTT. Plates were incubated for 4 h at 37 °C, before removal of the media and replacement with 1:1 (*v*/*v*) isopropanol:DMSO. Formation of formazan was quantified by color generation at a wavelength of 570 nm using a Multiskan Spectrum plate reader (Thermo Electron Corporation, Vantaa, Finland). Cell viability of cells treated with drugs was quantified by comparison to untreated control cells, set at 100% viability.

### 2.5. Statistical Analysis

Values generated for IC_50_ and for the dual-drug CI were determined using CompuSyn software. Cell viability data was calculated by non-linear regression (inhibitor vs. normalized response—variable slope) using a model of best-fit values of four data points using GraphPad Prism V.7 (https://www.graphpad.com/scientific-software/prism/). For cell viability assays, a one-way ANOVA was performed to determine significant changes after treatments compared to control cell viability, set at 100%.

## 3. Results

### 3.1. Drug Inhibition of AChE Protein In Vitro

**Figure 1:**
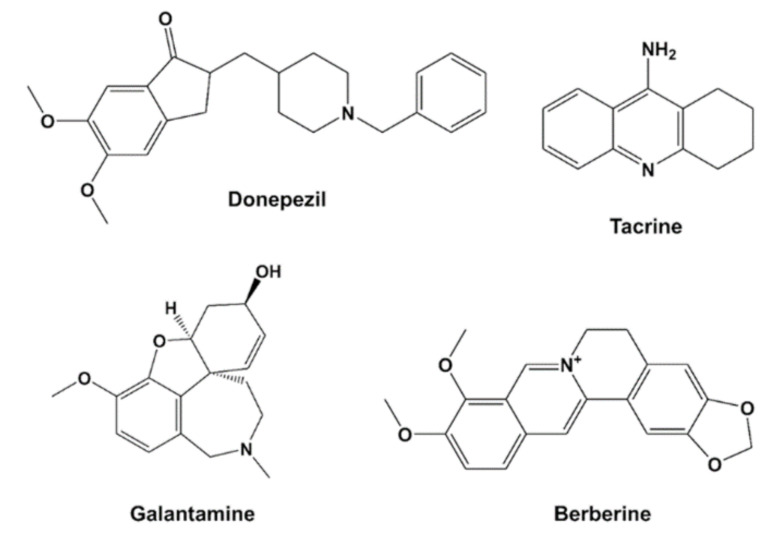
Structures of the cholinesterase inhibitor (ChEI) drugs.

**Figure 2:**
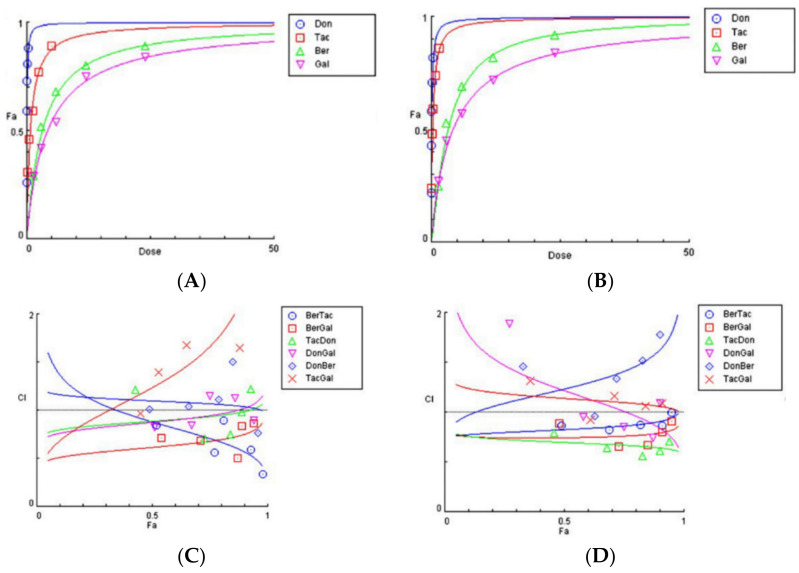
Drug inhibition of acetylcholinesterase (AChE) protein in vitro: Human AChE (**A**) or *Torpedo californica* AChE (**B**) were incubated with donepezil (Don), tacrine (Tac), berberine (Ber), and galantamine (Gal) and the level of cholinesterase inhibition quantified. Dose-response effect curves were generated as fractional affect (Fa) 0.5 (representing the concentration producing 50% enzyme inhibition (IC_50_) against dose. Combination index (CI) plots for dual-drug inhibition of human AChE (**C**) and *Torpedo californica* AChE (**D**). BerTac = berberine and tacrine; BerGal = berberine and galantamine; TacDon = tacrine and donepezil; DonGal = donepezil and galantamine; DonBer = donepezil and berberine; TacGal = tacrine and galantamine.

The drugs donepezil, tacrine, berberine, and galantamine (Figure 1) were incubated with recombinant human acetylcholinesterase (hAChE) and *Torpedo californica* acetylcholinesterase (*tc*AChE), and the level of enzymatic inhibition quantified. Dose-response effect curves were generated and a plot of fractional affect (Fa) 0.5 (representing the concentration producing 50% enzyme inhibition (IC_50_) against dose plotted (Figure 2A,B). The inhibition curves showed that the potency of the drugs to inhibit AChE was in the order: donepezil > tacrine > berberine > galantamine for both the human (h) and *Torpedo californica* (*tc*) enzymes. Berberine and galantamine inhibited both species of AChE with similar potency. Donepezil was more potent an inhibitor of human AChE, and tacrine was ≈2.7-fold more potent for the *T. californica* enzyme, as detailed in Table 1.

The ability of two-drug combinations to inhibit human and *tc*AChE were then considered. Mixtures of berberine and tacrine (BerTac), berberine and galantamine (BerGal), tacrine and donepezil (TacDon), donepezil and galantamine (DonGal), donepezil and berberine (DonBer), and tacrine and galantamine (TacGal) were assayed at combinations of 0.25×, 0.5×, 1×, 2×, and 4×, as detailed in Supplementary Table S1. A combination index (CI) plot (from CompuSyn) of the different drug combinations was generated for the fractional affect (Fa) 0.5 (IC_50_ values) (Figure 2C,D). The central line with a CI of 1 reflects the point at which drug combinations had an additive effect. CI values below that line are indicative of synergism (CI < 1), and CI values above the line represent antagonism (CI > 1) [31].

The drug combinations, BerTac, BerGal, and TacDon produced synergistic outcomes, whereas DonBer and TacGal were antagonistic. DonGal was synergistic for human AChE but antagonistic for *tc*AChE (Table 1). The most potent synergistic combination to inhibit human or *tc*AChE was from a berberine and galantamine mixture (BerGal). This was in stark contrast to their individual drug potencies that displayed higher IC_50_ values than either of the synthetic ChEIs, donepezil or tacrine. If BerGal was used at a 1:1 combination, IC_50_ values were lowered to 1.101 µM for hAChE inhibition and 1.355 µM for *tc*AChE (Table 2).

### 3.2. Drug Inhibition of AChE within Differentiated Neurons

**Figure 3:**
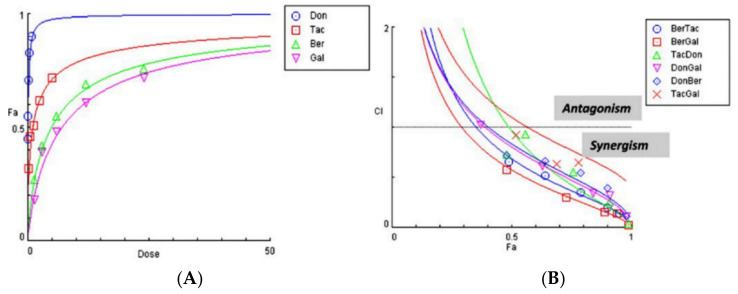
Drug inhibition of AChE within SH-SY5Y cells: Human neuroblastoma cells were incubated with donepezil (Don), tacrine (Tac), berberine (Ber), and galantamine (Gal) and the level of cholinesterase inhibition quantified (**A**). Combination index (CI) plot of dual-drug inhibition of AChE (**B**). BerTac = berberine and tacrine; BerGal = berberine and galantamine; TacDon = tacrine and donepezil; DonGal = donepezil and galantamine; DonBer = donepezil and berberine; TacGal = tacrine and galantamine.

Human derived neuroblastoma SH-SY5Y cells were differentiated with retinoic acid to produce neurons of a cholinergic phenotype [32,33,34,35], and then challenged with individual ChEIs and two-drug combinations, prepared according to Supplementary Table S1. Dose effect curves for the tested drugs were generated (Figure 3A), and showed that the relative drug potency observed for protein assays (Figure 2A,B) was mirrored with human neuronal cells grown in culture, with potency in the order donepezil > tacrine > berberine > galantamine. However, the cell-based assay curves were flatter and generated IC_50_ values higher than those obtained with purified hAChE (Table 2).

A CI plot of the different drug combinations against the fractional affect (Fa) 0.5 (IC_50_ values) revealed that for the first assayed point (0.25×), only TacDon, DonGal, and TacGal generated values close to the central line, in keeping with additive effects, whereas BerTac, BerGal, and DonBer effects were all synergistic. However, at higher drug concentrations, all of the tested combinations (BerTac, BerGal, TacDon, DonGal, DonBer, and TacGal) displayed synergistic effects (Figure 3B).

The CI values generated for the drug combinations are listed in Table 2, and show that the drug combinations BerTac, BerGal, DonGal, and DonBer all show synergistic inhibition of AChE at their IC_50_ concentrations. The TacDon drug combination approached additive effects, while TacGal was antagonistic at IC_50_ concentrations.

**Figure 4:**
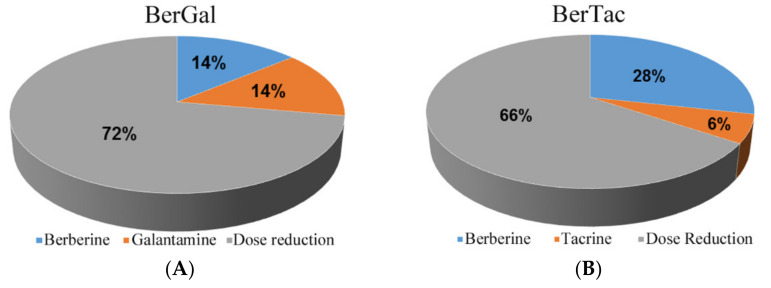
Virtual ChEI dual-drug combination pills: A virtual berberine and galantamine (BerGal) pill would have a 72% reduction of total drug dose (**A**). A virtual berberine and tacrine (BerTac) pill would have a 66% reduction of total drug dose (**B**).

The BerGal drug combination had the most potent inhibitory effect (CI = 0.575). The individual IC_50_ values for berberine and galantamine were 4.844 µM and 7.009 µM, respectively. Hence, a drug combination of 1.648 µM of each drug (IC_50_ values for a 1:1 drug ratio) would in theory have similar efficacy as either drug, but with a substantial reduction of dose: 34% of the original berberine and 24% of galantamine. Figure 4A is a pictorial representation of this virtual BerGal combination pill and has a net reduction of 72% of total drug dose.

Similarly, for the second most favorable drug combination of BerTac (CI = 0.686), there would be a beneficial reduction from their individual drug concentrations: IC_50_ values of 4.844 µM and 1.019 µM reduced to 1.670 µM and 0.348 µM, respectively. A theoretical BerTac pill would only require 28% and 6% of the individual doses of berberine and tacrine, respectively, with a net 66% reduction of total drug dose (Figure 4B).

### 3.3. Cytotoxicity of Individual and Dual-Drug ChEI Combinations to Neuronal Cells

**Figure 5:**
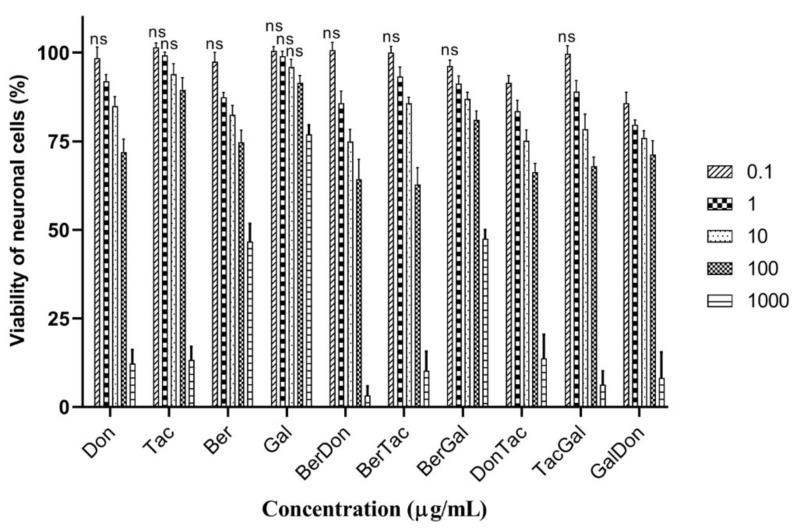
Cytotoxicity of individual and dual-drug ChEI combinations to SH-SY5Y cells: Human neuroblastoma cells were incubated with donepezil (Don), tacrine (Tac), berberine (Ber), and galantamine (Gal) or dual-drug combinations: BerTac (berberine and tacrine), BerGal (berberine and galantamine), TacDon (tacrine and donepezil), DonGal (donepezil and galantamine), DonBer (donepezil and berberine), and TacGal (tacrine and galantamine), and cell viability quantified using a MTT assay. Significant changes were quantified using a one-way ANOVA. ns = non-significant effect on cell viability. All other histograms not marked with ns, displayed significant changes from controls.

SH-SY5Y cells were treated with the ChEIs and two-drug combinations, and cell viability assessed using a MTT assay. Drugs were applied over a concentration range of 0.1, 1, 10, 100, and 1000 µg/mL for 48 h and cell viability compared with vehicle-treated control cells (set at 100% viability). The toxicity of the individual ChEIs to neuronal cells is shown in Figure 5. At the lowest concentration employed, 0.1 µM, none of the drugs were toxic to neurons (marked as non-significant changes from controls). A concentration of 1 µM (and above) of donepezil or berberine was able to induce toxicity, whereas at least 10 µM of tacrine or 100 µM of galantamine was needed to reduce cell viability. The drug (inhibitor) concentration that reduced cell viability by 50% (IC_50_) was determined by non-linear regression, with values listed in Table 3. The order of toxicity of the ChEIs was donepezil > tacrine > berberine > galantamine. Dual-drug combinations were generally more toxic to neuronal cells with lower IC_50_ values, apart from a BerGal combination that was relatively non-toxic (Figure 5 and Table 3).

## 4. Discussion

The drug treatment regimen for AD has not changed dramatically in over 20 years, with the current second-generation ChEIs, donepezil hydrochloride (FDA approved in 1997), rivastigmine tartrate (FDA approved in 2000), and galantamine hydrobromide (FDA approved in 2001), providing the mainstay treatment for mild-to-moderate AD. In addition, memantine (FDA approved in 2003), an antagonist of the N-methyl-D-aspartate (NMDA) receptor involved in excitatory glutamatergic neurotransmission, can also be used as a monotherapy, combination therapy, or adjunct to ChEI treatment. A drawback to the use of ChEIs is potential for ADRs as well as concerns regarding their cost-effectiveness [36,37]. Notably, tacrine, the first FDA approved ChEI (1993), was eventually removed as a prescription medication due to concerns with off-target toxicity effects, particularly hepatotoxicity [38]. To address this unmet need to improve dose efficiency, and thereby reduce the likelihood of induction of undesired side-effects, and also to provide a more cost-effective medicine, we examined the potential benefits associated with novel dual-drug ChEI combinations.

When assessed as single drugs, the ChEIs, donepezil, tacrine, berberine, and galantamine, all proved useful and potent inhibitors of human or *Torpedo californica* AChE. Donepezil was the most effective ChEI and then tacrine, with these two inhibitors producing IC_50_ values in the nanomolar range, similar to those published for rat brain homogenates [39]. Substrate (AChE) binding by a ChEI may be to a similar site [39], but AChE can potentially accommodate two ChEI molecules simultaneously. The catalytic region of AChE (the esteratic site) is located at the bottom of a narrow gorge and contains three essential amino acids that provide the catalytic triad [24,40]. Anti-cholinergic drugs can inhibit AChE via direct binding to the catalytic site or via binding to a peripheral anionic site (PAS) primarily composed of aromatic amino acids. A number of ChEIs, including donepezil, tacrine, berberine, and galantamine, can bind at the PAS, and binding at this site can contribute to reduced AChE activity [24,41,42,43,44].

Screening of human or *Torpedo californica* AChE inhibition with dual-drug combinations revealed that BerTac, BerGal, and TacDon produced synergistic outcomes. For human AChE present within differentiated neurons, dual-drug synergism was also evidenced for these combinations as well as from DonGal, DonBer, and TacGal. The most potent synergism was attained via the BerGal combination and then the BerTac pairing. The potential benefit of synergistic inhibition by BerGal was a 72% reduction in total drug dose and a 66% reduction with BerTac. Reduced drug load would be expected to have a concomitant reduction of potential ADRs as well as improved cost-effectiveness. This is particularly pertinent for tacrine, given that excessive ADRs were responsible for its removal from the drug market [36,38].

To assess potential neurotoxicity, cell viability of differentiated neurons was quantified as single drug and dual-drug combinations. For the single drug exposures, the order of potency as a ChEI, donepezil > tacrine > berberine > galantamine, was mirrored with corresponding drug toxicity to cells (Table 1 and Table 3). However, even for the most toxic drug, donepezil, (IC_50_ of 5.55 µM), the corresponding IC_50_ for AChE inhibition was only 0.081 µM (Table 2). Hence, therapeutic ChEI activity would still be efficacious without induction of significant neuronal toxicity.

Apart from BerGal, which was particularly well tolerated, each of the dual-drug combinations were of similar toxicity, and collectively, more toxic than donepezil (Table 3). Nevertheless, similar to application of individual drugs, the potency of dual-drug combinations towards AChE (IC_50_s) were approximately two to three orders of magnitude lower than that for cell viability IC_50_s; indicative of useful drug application within a therapeutic index.

The use of ChEIs for the treatment and management of AD may be improved through the use of dual-drug combinations or multi-drug targeting approaches [45,46,47,48]. The FDA approved concomitant use of memantine and ChEIs has primarily proved clinical efficacious and comparable to monotherapies, and for some studies provided additional benefit of reduced neurobehavioral symptoms [48].

In summary, there is an ongoing requirement for utilization of ChEIs with similar or improved clinical efficacy but with reduced ADRs. In the absence of newly available prescription drugs, deployment of current ChEIs (donepezil or galantamine), redeployment of a past ChEI (tacrine), or employment of a new ChEI (berberine) as dual-drug combinations, represent a potential means to achieve drug efficacy at lower drug doses able to diminish toxicity and increase drug cost-effectiveness. However, although dual-drug combinations are able to induce synergistic inhibition of AChE, individual drug pharmacokinetic profiles may yet hamper therapeutic utilization. For example, only donepezil and galantamine have relatively high oral bioavailability (compared to tacrine or berberine), and only the former drug has a half-life of over 24 h, facilitating a single daily dosing regimen [11,49,50,51]. Hence, in vivo studies are required to validate these in vitro results and the potential for implementation of coordinated dual-drug ChEI treatments for AD.

## Figures and Tables

**Table 1:** Quantitation of interactions between ChEI drugs, dual-drug combinations, and human and *Torpedo californica* AChE.

Drug or Drug Combination	CI Value	Acetycholinesterase Inhibition (IC_50_) (nM)			
		Donepezil	Tacrine	Berberine	Galantamine
Don		57 (h)			
		91 (tc)			
Tac			726 (h)		
			271 (tc)		
Ber				3131 (h)	
				3234 (tc)	
Gal					4183 (h)
					4173 (tc)
BerTac	0.880 (h)		302 (h)	1451 (h)	
	0.826 (tc)		99 (tc)	1486 (tc)	
BerGal	0.615 (h)			1101 (h)	1101 (h)
	0.744 (tc)			1355 (tc)	1355 (tc)
TacDon	0.887 (h)	27 (h)	299 (h)		
	0.697 (tc)	29 (tc)	103 (tc)		
DonGal	0.876 (h)	29 (h)			1538 (h)
	1.216 (tc)	51 (tc)			2729 (tc)
DonBer	1.099 (h)	32 (h)		1690 (h)	
	1.233 (tc)	45 (tc)		2393 (tc)	
TacGal	1.235 (h)		489 (h)		2349 (h)
	1.139 (tc)		156 (tc)		2344 (tc)

Don = Donepezil; Tac = tacrine; Ber = berberine; Gal = galantamine. BerTac = berberine and tacrine; BerGal = berberine and galantamine; TacDon = tacrine and donepezil; DonGal = donepezil and galantamine; DonBer = donepezil and berberine; TacGal = tacrine and galantamine. (h) = human; tc = *Torpedo californica*. Combination Index values (CI): Synergism: CI < 1; Additive: CL = 1; Antagonism: CI > 1.

**Table 2:** Quantitation of interactions between ChEI drugs, dual-drug combinations, and neuronal AChE.

Drug or Drug Combination	CI Value	Acetylcholinesterase Inhibition (IC_50_) (nM)			
		Donepezil	Tacrine	Berberine	Galantamine
Don		81			
Tac			1019		
Ber				4844	
Gal					7009
BerTac	0.686		348	1670	
BerGal	0.575			1648	1648
TacDon	0.963	41	461		
DonGal	0.804	40			2152
DonBer	0.834	36		1907	
TacGal	1.100		660		3169

Don = donepezil; Tac = tacrine; Ber = berberine; Gal = galantamine. BerTac = berberine and tacrine; BerGal = berberine and galantamine; TacDon = tacrine and donepezil; DonGal = donepezil and galantamine; DonBer = donepezil and berberine; TacGal = tacrine and galantamine. (h) = human; tc = *Torpedo californica*. Combination index values (CI); Synergism: CI < 1; Additive: CI = 1; Antagonism: CI > 1.

**Table 3:** Neurotoxicity of ChEI drugs and dual-drug combinations against SH-SY5Y neuroblastoma cells.

Drug or Drug Combination	Cell Cytotoxicity Log IC_50_ (µg/mL)
Don	2.31 ± 0.057
Tac	2.53 ± 0.091
Ber	2.98 ± 0.010
Gal	4.10 ± 0.041
DonBer	2.01 ± 0.043
BerTac	2.16 ± 0.031
BerGal	3.01 ± 0.046
TacDon	2.08 ± 0.038
TacGal	2.16 ± 0.052
DonGal	2.08 ± 0.059

## Data Availability

The data presented in this study are available from the authors upon request.

## Supplementary Materials

The following are available online at https://www.mdpi.com/2076-3425/11/2/184/s1, Table S1: Constant ratio experimental design for two-drug combinations.
