# Supplementary material for: Anti-Cholinesterase Combination Drug Therapy as a Potential Treatment for Alzheimer’s Disease
Source: Brain Sci. 2021 Feb 2;11(2):184. doi: 10.3390/brainsci11020184 (PMC7913148; doi:10.3390/brainsci11020184)
Supplement: Supplementary file 1 [file brainsci-11-00184-s001.pdf]

Supplementary Table S1: Constant ratio experimental design for two-drug combinations.

		Drug 1					
		**0**	**0.25X $(ED_{50})_1$**	**0.5X $(ED_{50})_1$**	**X $(ED_{50})_1$**	**2X $(ED_{50})_1$**	**4X $(ED_{50})_1$**
**Drug 2**	**0**	**Control $(fa)_0$**	**$(fa)_1$**	**$(fa)_1$**	**$(fa)_1$**	**$(fa)_1$**	**$(fa)_1$**
	**0.25X $(ED_{50})_2$**	**$(fa)_2$**	**$(fa)_{1,2}$**				
	**0.5X $(ED_{50})_2$**	**$(fa)_2$**		**$(fa)_{1,2}$**			
	**X $(ED_{50})_2$**	**$(fa)_2$**			**$(fa)_{1,2}$**		
	**2X $(ED_{50})_2$**	**$(fa)_2$**				**$(fa)_{1,2}$**	
	**4X (ED50)$_2$**	**$(fa)_2$**					**$(fa)_{1,2}$**

(fa), fractional affect; ED_{50}, effective dose producing a 50% effect
